# Body mass index in early and middle adult life: prospective associations with myocardial infarction, stroke and diabetes over a 30-year period: the British Regional Heart Study

**DOI:** 10.1136/bmjopen-2015-008105

**Published:** 2015-09-15

**Authors:** Christopher G Owen, Venediktos V Kapetanakis, Alicja R Rudnicka, Andrea K Wathern, Lucy Lennon, Olia Papacosta, Derek G Cook, S Goya Wannamethee, Peter H Whincup

**Affiliations:** 1 Population Health Research Institute, St George's, University of London, London, UK; 2 Department of Primary Care and Population Health, UCL Medical School, London, UK

**Keywords:** EPIDEMIOLOGY, STROKE MEDICINE

## Abstract

**Objectives:**

Adiposity in middle age is an established risk factor for cardiovascular disease and type 2 diabetes; less is known about the impact of adiposity from early adult life. We examined the effects of high body mass index (BMI) in early and middle adulthood on myocardial infarction (MI), stroke and diabetes risks.

**Design:**

A prospective cohort study.

**Participants:**

7735 men with BMI measured in middle age (40–59 years) and BMI ascertained at 21 years from military records or participant recall.

**Primary and secondary outcome measures:**

30-year follow-up data for type 2 diabetes, MI and stroke incidence; Cox proportional hazards models were used to examine the effect of BMI at both ages on these outcomes, adjusted for age and smoking status.

**Results:**

Among 4846 (63%) men (with complete data), a 1 kg/m^2^ higher BMI at 21 years was associated with a 6% (95% CI 4% to 9%) higher type 2 diabetes risk, compared with a 21% (95% CI 18% to 24%) higher diabetes risk for a 1 kg/m^2^ higher BMI in middle age (hazard ratio (HR) 1.21, 95% CI 1.18 to 1.24). Higher BMI in middle age was associated with a 6% (95% CI 4% to 8%) increase in MI and a 4% (95% CI 1% to 7%) increase in stroke; BMI at 21 years showed no associations with MI or stroke risk.

**Conclusions:**

Higher BMI at 21 years of age is associated with later diabetes incidence but not MI or stroke, while higher BMI in middle age is strongly associated with all outcomes. Early obesity prevention may reduce later type 2 diabetes risk, more than MI and stroke.

**Strengths and limitations of this study**

- This investigation is based on a geographically and socially representative cohort study with high response rates and exceptionally high follow-up rates.
- Linkage of military record data to this established middle-aged cohort provides novel information on early life exposures.
- The limited number of study participants with high levels of body mass index (BMI) in early life will have limited the power of the study for detection of the effects of BMI in early adult life on risks of myocardial infarction and stroke, though they were detectable for diabetes.
- Effects of high early BMI on diabetes may be potentially reversible by subsequent weight loss.
- Findings have important implications for type 2 diabetes prevention.

## Introduction

Increased adiposity in middle age and later life is an important risk factor for type 2 diabetes and cardiovascular disease, especially coronary heart disease (CHD).^1–5^ There has been a worldwide increase in the prevalences of overweight and obesity in recent decades, particularly in higher income countries.^6–8^ However, overweight and obesity are increasingly affecting younger as well as older people.^7^ Since high levels of adiposity track strongly from childhood and early adulthood to middle age, most people who become overweight in early adult life will be overweight or obese in middle age.^9^ ^10^ There is growing concern about the long-term health consequences of increased exposure to adiposity from early adult life, which may exacerbate the risks of cardiovascular disease and type 2 diabetes in later life.^11^ ^12^

To understand the consequences of adiposity over the life course requires studies with accurate data on adiposity at several points of the life course, including early adult life, middle age and in later life, with extended follow-up for relevant outcomes.^12^ ^13^ Birth cohort studies often have information on adiposity both before and during middle age,^14–16^ but generally have populations which are too young to have large numbers of chronic disease events in later life. Many prospective studies have data on adiposity in middle age and its relation to subsequent chronic disease outcomes,^2^ but few of these also have information on adiposity in early adult life.^12^

We have therefore used a novel approach to provide new information on this issue, obtaining data on measured weight and height in early adult life (at age 21 years) from military service records for participants in an established cohort of almost 8000 men in whom body mass index (BMI) has been studied in detail from middle age (40–59 years) onwards, and for whom at least 30 years of follow-up data on cardiovascular outcomes and diabetes are available. For the purpose of this report, BMI in early adult life (mean age 21 years) and middle age (mean age 50 years) will be referred to as BMI-21 and BMI-50.

## Methods

### The British Regional Heart Study

The British Regional Heart Study (BRHS) is a prospective study of cardiovascular disease, and type 2 diabetes among middle-aged and older British men. It is based on 7735 men born between 1919 and 1939 who were recruited in 1978–1980 aged 40–59 years from a single general practice in 24 British towns (78% response rate),^17^ and were followed up until the present. Study men completed a detailed questionnaire on entry to the study (including information on pre-existing cardiovascular disease, type 2 diabetes and other medical conditions, smoking status, physical activity, alcohol intake and social class) and had measurements of weight and height, measured with participants in light clothing without shoes. Weight was measured to the last 0.1 kg using regularly calibrated scales and height to the last complete 0.1 cm using a Harpenden stadiometer. Participants then completed periodic postal questionnaires about their health in 1983–1985, 1992 and 1996; in 1996, they were asked to recall their weight at age 21 years. Men have been followed up over a 30-year period for mortality, morbidity and disability with <2% loss to follow-up.

### Follow-up

Study participants were followed prospectively from the baseline examination at 40–59 years for all-cause mortality, cardiovascular morbidity and diabetes incidence from baseline to June 2010. Those with a doctor diagnosis of myocardial infarction (MI; n=134), stroke (n=19) and diabetes (n=50) at baseline (n=195 in all) were excluded. Follow-up has been achieved for 98% of the cohort.^17^ Information on deaths was collected through National Health Service Central Registers; death certificates were coded using the International Classification of Diseases, Ninth Revision (ICD-9). Fatal MI was defined as ICD-9 codes 410–414 and fatal stroke as ICD-9 codes 430–438. Information on non-fatal MI and stroke events was obtained according to ongoing general practitioner reports and two-yearly medical record reviews.^17^ A non-fatal MI was diagnosed according to WHO criteria.^18^ Non-fatal stroke events were those that produced a neurological deficit that was present for more than 24 h. New diabetes diagnoses (including date) were also obtained from medical record reviews.

### Obtaining information on weight and height in early adult life

Since most men in the BRHS cohort (born 1919–1939) would have undertaken military service in early adulthood, either during World War II (1939–1945) or during UK National Service (1945–1963), we sought information on weight in early adult life from military service records. In 2007, a questionnaire was sent to all surviving men enquiring about military service in early adulthood, including details of service period and service number where available, and permission was sought to obtain service medical records (archived at TNT Ltd, Swadlincote, Derbyshire). We have used this data source to obtain measurements of height and weight, which were recorded at service entry (in both service and medical records) and formed part of a standard military medical classification.^19^

### Statistical methods

We have previously outlined the methodology used to obtain weight at 21 years.^20^ In brief, when a record of weight measurement was recorded in service records between 20 and 22 years, this was used directly as an estimate of weight at 21 years. In the absence of measured weight between 20 and 22 years, a weight recorded between either 17 and 19 or 23 and 25 years was used to estimate weight at 21 years from a multilevel model fitted using all weight measurements recorded, adjusting for age (allowing for a quadratic relationship) and period of enlistment; including pre-World War II (1934–1938), War (1939–1945) and post-War periods (1946–1950, 1951–1955, 1956–1975). The model included a random intercept allowing for clustering of measures within individual, and a random slope for the linear term of age allowing person-specific age slopes to be determined. Where measured weight was not recorded, recalled weight in 1996 was used, adjusted for recall bias quantified by comparisons of measured and recalled weight at 21 years (available in 694 individuals). Modest overestimation of true weight among thinner men was observed (mean estimated bias across the range of measured weight was 1.5 kg with a maximum of 6.9 kg estimated at the lowest limit of the range of measured weight; see online supplementary figure S1). Height measurements recorded in military records were used to provide an estimate of height at 21 years using similar methods as for weight. BMI at 21 years was calculated as weight/height squared in kg/m^2^.

The exact dates of first MI (fatal or non-fatal), first stroke (fatal or non-fatal) and diagnosis of diabetes were recorded over a 30-year follow-up period from study entry. Incidence rates for all outcomes were summarised by quintiles of BMI-21 to examine trends. A Cox proportional hazards model was used to assess the separate effects of BMI-21 and BMI-50 on the risks of all outcomes (adjusting for age and smoking status at baseline, and town as a fixed effect). Additionally, the effect of BMI-50 on the risk of each outcome was stratified by quintiles of BMI-21 to examine evidence for effect modification. Metaregression of regression coefficients from the stratified analysis were used to examine tests for trend. The effect of BMI on each outcome was also examined using a life course approach, by using trajectories of exposure to high BMI at the two ages (BMI-21 and BMI-50). A high BMI was defined when an individual was on/above the 75th centile of BMI distribution at a specific age. This provided four mutually exclusive trajectories to examine the life course patterning of disease risk, adjusting for town (as fixed effect), age and smoking status.

## Results

In all, 4842 men (63% of the original cohort) had data on height and weight at both ages (ie, BMI-21 and BMI-50), smoking at BRHS baseline and 30 years of follow-up data for type 2 diabetes, MI and stroke. Weight data at 21 years of age were based on military records for 2205 (44%) men; measured weight at age 20–22 years was obtained directly for 1258 men and imputed from weights between 17 and 19 years or between 23 and 25 years of age for 947 men. Weight at age 21 years was recalled from middle age for the remaining 2832 men. A subset of 694 men with both recalled and measured weight in early adult life allowed bias in recalled weight to be quantified; modest overestimation of true weight among thinner men was observed (see online supplementary figure S1). Similar numbers were obtained for height at 21 years of age, with 1244 men measured between 20 and 22 years of age, 963 measured between 17 and 19 or 23 and 25 years and with 2830 imputed from middle age (allowing for a very small amount of shrinkage). Mean BMI-50 was only marginally higher among the 2890 men excluded from analysis compared with those included (25.6 vs 25.4 kg/m^2^, p=0.025).

Table 1 shows the anthropometric characteristics of the cohort at 21 and 50 years of age, overall and by quintiles of BMI in early adulthood. Weight and BMI at 50 years increased with higher quintiles of BMI in early adult life (BMI-21). Moreover, prevalences of overweight (defined as BMI ≥25 kg/m^2^) and obesity (defined as BMI ≥30 kg/m^2^) were considerably higher at 50 years (46%, 7.3%) compared with prevalence at 21 years (9.6%, 1.5%). Height remained largely stable between early adult life and middle age with a minimal amount of shrinkage.

**Table 1:** Cohort characteristics in early (21 years) and middle adulthood (50 years) by quintile of BMI in early adulthood

Characteristic			Quintile group of BMI in early adulthood									
	All		0–20%		21–40%		41–60%		61–80%		81–100%	
	N	Mean (SD)	N	Mean (SD)	N	Mean (SD)	N	Mean (SD)	N	Mean (SD)	N	Mean (SD)
At 21 years												
Weight (kg)	4842	66.4 (10.0)	963	55.8 (6.2)	971	62.1 (4.4)	971	65.5 (4.5)	965	69.2 (5.3)	972	79.1 (9.9)
Height (m)	4842	1.74 (0.06)	963	1.74 (0.06)	971	1.74 (0.06)	971	1.74 (0.06)	965	1.74 (0.06)	972	1.74 (0.06)
BMI (kg/m^2^)	4842	21.9 (2.9)	963	18.4 (1.4)	971	20.5 (0.4)	971	21.7 (0.3)	965	22.9 (0.4)	972	26.0 (2.5)
At 50 years												
Age (years)	4842	49.6 (5.7)	963	50.2 (5.8)	971	50.0 (5.7)	971	49.7 (5.7)	965	49.3 (5.6)	972	48.7 (5.7)
Weight (kg)	4842	76.7 (10.7)	963	71.9 (9.9)	971	73.5 (9.1)	971	75.7 (9.5)	965	78.3 (9.8)	972	84.0 (10.8)
Height (m)	4842	1.74 (0.07)	963	1.74 (0.07)	971	1.74 (0.06)	971	1.73 (0.06)	965	1.73 (0.07)	972	1.74 (0.07)
BMI (kg/m^2^)	4842	25.4 (3.1)	963	23.8 (2.9)	971	24.4 (2.6)	971	25.1 (2.7)	965	26.0 (2.7)	972	27.7 (3.0)

Quintiles of BMI in early adulthood are calculated using all available data.

BMI, body mass index; N, number of participants.

Table 2 summarises the incidence rates for diabetes, MI and stroke (fatal and non-fatal) within the cohort overall, and by quintiles of BMI-21 and BMI-50. While there was no evidence of a trend in incidence rates for MI or stroke (fatal and non-fatal) across quintiles of BMI-21, incidence rates of diabetes appeared higher among men who were in the upper quintile of BMI (the 81st–100th centile) in early life. Incidence rates for diabetes, MI (fatal and non-fatal) and non-fatal stroke appeared positively associated with quintiles of BMI-50.

**Table 2:** Summary statistics for survival time data for all outcomes by quintile of BMI-21 and BMI-50

	Diabetes				MI				Stroke				Fatal MI				Fatal stroke			
	n/N	prys	r	Mean event age (SD)	n/N	prys	r	Mean event age (SD)	n/N	prys	r	Mean event age (SD)	n/N	prys	r	Event age Mean (SD)	n/N	prys	r	Mean event age (SD)
All	627/4842	124 911	4.8	67.9 (8.3)	976/4842	124 249	7.6	68.2 (9.6)	547/4842	127 529	4.1	71.3 (8.7)	552/4842	131 069	3.6	73.0 (8.2)	173/4842	131 069	1.0	76.7 (7.1)
Quintiles of BMI-21																				
0–20%	134/963	24 955	5.2	68.0 (8.4)	212/963	24 738	7.9	69.1 (9.9)	121/963	25 678	4.3	73.2 (8.6)	116/963	26 330	3.4	74.6 (7.7)	37/963	26 330	1.0	77.8 (6.5)
21–40%	111/971	24 897	4.4	69.0 (7.9)	207/971	24 429	8.0	68.9 (9.3)	121/971	25 049	4.5	70.7 (8.2)	118/971	25 910	3.7	74.5 (7.8)	43/971	25 910	1.2	77.2 (6.0)
41–60%	105/971	25 061	4.0	67.9 (8.1)	184/971	24 763	7.2	67.1 (8.9)	111/971	25 360	4.1	71.1 (8.8)	101/971	26 075	3.3	71.1 (7.7)	38/971	26 075	1.1	76.5 (9.1)
61–80%	111/965	24 823	4.3	68.3 (7.9)	177/965	24 627	7.0	66.9 (9.6)	96/965	25 296	3.7	71.4 (8.8)	101/965	25 868	3.4	71.6 (8.9)	32/965	25 868	1.0	75.7 (6.4)
81–100%	166/972	25 175	6.3	66.6 (8.9)	196/972	25 692	7.7	68.7 (9.8)	98/972	26 146	3.8	69.7 (8.8)	116/972	26 886	4.0	72.6 (8.4)	23/972	26 886	0.7	75.6 (7.0)
Quintiles of BMI-50																				
0–20%	49/979	26 088	1.8	69.9 (7.9)	174/979	25 319	6.4	67.7 (9.6)	96/979	25 845	3.6	70.7 (8.9)	102/979	26 482	3.2	71.7 (8.6)	33/979	26 482	0.9	76.1 (7.2)
21–40%	83/997	26 573	3.0	69.0 (7.8)	178/997	26 028	6.9	69.2 (9.3)	107/997	26 662	4.0	71.7 (9.2)	90/997	27 319	2.9	74.5 (7.9)	39/997	27 319	1.1	76.3 (8.5)
41–60%	114/980	25 809	4.2	68.2 (8.1)	182/980	25 663	7.0	68.0 (10.1)	115/980	26 264	4.2	71.8 (8.7)	104/980	26 956	3.3	73.1 (8.1)	36/980	26 956	1.0	77.6 (6.3)
61–80%	148/982	25 125	5.7	68.2 (8.4)	205/982	24 986	7.7	68.6 (9.5)	113/982	25 680	4.0	71.6 (8.7)	112/982	26 484	3.5	73.7 (8.6)	31/982	26 484	0.8	79.1 (5.9)
81–100%	233/904	21 316	10.6	66.6 (8.5)	237/904	22 253	10.2	67.7 (9.3)	116/904	23 078	4.7	70.6 (8.1)	144/904	23 829	5.1	72.2 (7.6)	34/904	23 829	1.0	74.5 (6.5)

BMI-21 quintiles calculated using all available data.

BMI, body mass index; MI, myocardial infarction; n, number of cases; N, total number of participants; pyrs, person years at risk; r, incidence rate reported as number of new cases per 1000 individuals per year, adjusted for smoking status, town (fixed effect), standardised to age 50.

Table 3 shows hazard ratios (HRs) for each outcome per unit increase in BMI (per 1 kg/m^2^), separately at 21 and 50 years of age. A 1 kg/m^2^ higher BMI-21 was associated with a 6% higher risk for diabetes in later life (HR 1.06, 95% CI 1.04 to 1.09), but not with a higher MI or stroke risk. BMI-50 was strongly associated with higher diabetes risk (HR 1.21, 95% CI 1.18 to 1.24)—three times the effect size for BMI-21—and also with higher risks of MI (HR 1.06, 95%CI 1.04 to 1.08) and stroke (HR 1.04, 95% CI 1.01 to 1.07). Fatal MI and fatal stroke showed patterns broadly similar to those for all fatal and non-fatal MI and stroke events, respectively.

**Table 3:** HRs for all outcomes per 1 kg/m^2^ higher BMI at mean ages 21 and 50 years

Outcome	BMI-21			BMI-50		
	N	HR (95% CI)	p Value	N	HR (95% CI)	p Value
Diabetes	4842	1.06 (1.04 to 1.09)	<0.001	4842	1.21 (1.18 to 1.24)	<0.001
MI	4842	0.99 (0.97 to 1.02)	0.63	4842	1.06 (1.04 to 1.08)	<0.001
Stroke	4842	0.99 (0.96 to 1.02)	0.48	4842	1.04 (1.01 to 1.07)	0.010
Fatal MI	4842	1.01 (0.98 to 1.04)	0.35	4842	1.07 (1.04 to 1.10)	<0.001
Fatal stroke	4842	0.97 (0.92 to 1.03)	0.29	4842	1.02 (0.97 to 1.07)	0.45

HR for the effect of a 1 kg/m^2^ increase in BMI, adjusted for age, smoking at 50 years of age and town (fixed effect).

BMI, body mass index; MI, myocardial infarction; N, total number of participants.

Table 4 shows the associations between life course BMI trajectories on cardiometabolic outcomes in later life, expressed as HRs from the reference trajectory (0–0), that is, participants with low levels of BMI at both 21 and 50 years of age. High BMI-50 with or without high BMI-21 was associated with approximately a threefold higher risk of diabetes, and a 30–60% higher risk of MI (including fatal MI). High BMI-21 with high BMI-50 was associated with a marked increase in diabetes risk and to a lesser extent in MI; high BMI-21 without BMI-50 was not associated with increased risks of diabetes or cardiovascular disease.

**Table 4:** HRs for all outcomes by patterns of high BMI at mean ages 21 and 50 years

BMI trajectory	N	BMI-21 mean (SD)	BMI-50 mean (SD)	Diabetes HR (95% CI)	MI HR (95% CI)	Stroke HR (95% CI)	Fatal MI HR (95% CI)	Fatal HR (95% CI)
0–0	3055	20.6 (1.7)	23.9 (2.1)	1	1	1	1	1
1–0	637	24.9 (1.7)	25.2 (1.6)	0.81 (0.60 to 1.08)	0.95 (0.78 to 1.17)	0.81 (0.62 to 1.08)	1.05 (0.81 to 1.38)	0.65 (0.38 to 1.12)
0–1	574	21.2 (1.9)	29.3 (2.0)	3.02 (2.45 to 3.73)^a^	1.63 (1.37 to 1.95)^a^	1.25 (0.98 to 1.60)	1.80 (1.44 to 2.26)^a^	1.12 (0.72 to 1.74)
1–1	576	26.2 (2.9)	29.9 (2.2)	2.84 (2.32 to 3.48)^a^	1.28 (1.06 to 1.56)^b^	1.01 (0.77 to 1.32)	1.51 (1.17 to 1.94)^b^	0.92 (0.56 to 1.53)

For BMI-21 and BMI-50, this includes all available data.

BMI trajectories: the values 0 and 1 denote BMI below and above the 75th centile, respectively. Each couple of zeros and ones corresponds to different trajectories of high BMI in early and middle adulthood.

HR comparing the risk of cardiovascular outcome in different trajectories of BMI with the 0–0 reference group. Estimates are adjusted for age and smoking in middle adulthood and town (fixed effect).

^a^p<0.001, ^b^p<0.05.

BMI, body mass index; MI, myocardial infarction; N, total number of participants.

The influence of BMI-21 on the associations between BMI-50 and each outcome is summarised in table 5. The risk of diabetes associated with high BMI-50 appeared slightly weaker among men in the lowest quintile of BMI-21, but there was no strong evidence of interaction (p=0.19 for difference in effect of BMI-50 by quintile of BMI-21—table 5). The associations between BMI-50, MI and stroke did not differ appreciably across quintiles of BMI-21. A sensitivity analysis performed by analysing the first 15 years of follow-up after BRHS baseline yield similar results (data not presented). Moreover, associations were not materially altered by additional adjustment for social class (data not presented).

**Table 5:** HRs for the associations between BMI at mean age 50 years and all outcomes, stratified by BMI at mean age 21 years

	Diabetes		MI		Stroke		Fatal MI		Fatal stroke	
	N	HR (95% CI)	N	HR (95% CI)	N	HR (95% CI)	N	HR (95% CI)	N	HR (95% CI)
Overall BMI	4842	1.21 (1.18 to 1.24)^a^	4842	1.06 (1.04 to 1.08)^a^	4842	1.04 (1.01 to 1.07)^b^	4842	1.07 (1.04 to 1.10)^a^	4842	1.02 (0.97 to 1.07)
BMI-21 quintiles										
0–20%	963	1.17 (1.11 to 1.23)^a^	963	1.06 (1.02 to 1.11)^b^	963	1.04 (0.98 to 1.11)	963	1.06 (0.998 to 1.13)	963	1.07 (0.95 to 1.21)
21–40%	971	1.30 (1.20 to 1.40)^a^	971	1.08 (1.02 to 1.14)^b^	971	1.03 (0.96 to 1.11)	971	1.07 (0.99 to 1.15)	971	0.93 (0.82 to 1.06)
41–60%	971	1.24 (1.16 to 1.32)^a^	971	1.10 (1.04 to 1.16)^a^	971	1.09 (1.02 to 1.17)^b^	971	1.12 (1.05 to 1.21)^b^	971	1.04 (0.91 to 1.18)
61–80%	965	1.29 (1.21 to 1.38)^a^	965	1.11 (1.05 to 1.17)^a^	965	1.05 (0.97 to 1.14)	965	1.07 (0.99 to 1.15)	965	1.07 (0.93 to 1.23)
81–100%	972	1.27 (1.21 to 1.34)^a^	972	1.06 (1.01 to 1.11)^b^	972	1.06 (0.998 to 1.14)	972	1.09 (1.03 to 1.16)^b^	972	1.09 (0.97 to 1.23)
p trend		0.19		0.82		0.62		0.63		0.47

HR for the effect of a 1 kg/m^2^ increase in BMI in middle adulthood, adjusted for age, smoking status in middle life and town (fixed effect).

^a^p<0.001, ^b^p<0.05.

BMI, body mass index; MI, myocardial infarction; N, number of participants; p trend, p value for trend across groups regarding the effect of BMI-50 (obtained from metaregression).

## Discussion

### Main findings

Adiposity in middle age is a well-known risk factor for cardiovascular disease and type 2 diabetes.^2^ However, less is known about the impact of high BMI from early adult life on these outcomes, as long-term cohort studies (particularly from birth) rarely have follow-up to an age when cardiometabolic outcomes readily occur. This study provides important insight into the role of BMI, as a measure of adiposity in early adult life on cardiovascular and diabetes risks from middle age. BMI in early adult life was independently associated with later diabetes risk, but not with MI and stroke risks. BMI in middle age was more strongly associated with type 2 diabetes, and was also associated with MI and stroke risks.

### Relation to earlier studies

Our findings are coherent with established evidence showing that adiposity in middle age shows strong, graded associations, most strongly with type 2 diabetes but also with CHD and stroke.^2^ ^4^ ^5^ ^11^ ^21^ The finding that BMI in early adult life is associated with the subsequent incidence of diabetes is consistent with recent prospective reports linking higher levels of adiposity in early adult life to diabetes risks^22–25^ and with recent reports suggesting that longer duration of adiposity (particularly from early adult life) is related to diabetes risk.^26–29^ It is also consistent with reported associations between BMI in early adult life and insulin resistance and glycaemia, both in the present study^20^ and in other cohorts.^30^ This is also compatible with a recent large study of Israeli army recruits, which showed that high BMI in adolescence was strongly related to metabolic disorders in later life.^24^ This study also showed that adolescents with higher BMI who become lean in adulthood, do not have an increase and may have a reduction in their risk of diabetes.^24^ This is consistent with our finding that men who were overweight at 21 but not at 50 had similar or possibly lower levels of diabetes than those who were not overweight at any time, and with experimental evidence showing that weight loss has a marked effect on diabetes risk.^31^

Earlier studies and reviews have suggested that higher levels of adiposity in adolescence and early adult life may be associated with risks of CHD,^12^ ^32^ ^33^ at variance with our findings for MI and stroke here. However, the independence of the early BMI-CHD associations has not so far been confirmed.^12^ ^32^ Moreover, in the present study, there were few participants with high levels of body fatness in early adult life, limiting the power of the study to detect associations between early BMI and cardiovascular disease, which would be expected to be weaker than those for diabetes. While BMI has been strongly correlated with total and visceral body fat in previous studies of adults, it should be acknowledged that BMI as a measurement of adiposity in these young men may be more closely related to lean muscle mass than to fat mass.^34^ ^35^ It is also possible that selection bias may have influenced the findings, particularly the possibility that men with high BMI-21 levels developed CHD in early middle age, making their inclusion in the study less likely. However, the marginally higher BMI-50 among men excluded from analysis compared with those included (approximately 0.2 kg/m^2^), and consistency of associations between early BMI and CHD risk between 15 and 30 years of follow-up makes this less likely.

### Strengths and limitations

This investigation is based on a geographically and socially representative cohort study with high response rates and exceptionally high follow-up rates. Among men who took part in the baseline examination, it was possible using two new sources of information, military health records and participant recall, to obtain information on BMI at 21 years for almost two-thirds of study participants (65%). Information from military records was documented methodically at the time of measurement and would be expected to have a high degree of validity. Among men with data from both sources, recalled data were substantially consistent with military record data, and allowed adjustment for a small bias in recalled weight. Although the analyses were based on two-thirds of the original cohort, and therefore being based on a healthy survivor group, baseline BMI and cardiovascular risk factor profiles in men who were or were not included in the present analyses did not differ markedly, suggesting that selection bias is unlikely to have led to underestimation of potential associations between BMI in early adult life and later risks of diabetes and cardiovascular disease. This is consistent with other evidence suggesting that selection bias is unlikely to have a marked influence on exposure–outcome associations.^36^ However, the limited number of study participants with high levels of BMI in early life will have limited the power of the study for detection of the effects of BMI in early adult life on risks of MI and stroke, though they were detectable for diabetes. A further limitation of the study is the absence of information on weight between age 21 years and study entry (between 40 and 59 years) making accurate determination of duration of adiposity between early adult life and middle age infeasible.

The analytic approaches taken in this report correspond with those in other recent studies^37–40^ in avoiding adjustment for adiposity levels at different ages; results based on mutual adjustment for adiposity measures at different stages of the life course (which are highly intercorrelated) are likely to be misleading.^32^ ^41^ ^42^ The approach taken has therefore focused on the comparative strength and consistency of independent associations between BMI at each age time point (in early and middle age) and cardiometabolic outcomes in later life. Moreover, we have assessed how different patterns and potentially cumulative/combined effects of high BMI in early and middle age impact on cardiometabolic outcomes. Our earlier work has demonstrated the potential for confusion when reparameterising statistical models examining the effect of life course BMI on disease outcomes.^20^

While findings from this study are likely to be generalisable to middle-aged and older white men, relevance to women or a similar age (who show different patterns and trends in body composition, eg, greater losses in muscle mass with age), and other ethnic groups remains to be established. BMI is only a proxy measure of adiposity, and high levels of BMI may not fully capture those that are overweight or obese, especially in historic cohorts. Moreover, the degree to which BMI captures true adiposity differs with age, as ageing is associated with significant changes in body composition.^43^

## Conclusion

This study provides novel and potentially important information on the role of BMI in early adult life on long-term cardiovascular and diabetes risk, based on the linkage of military service records from early adult life to an established cohort recruited in middle age. The results suggest that BMI in early adult life may have an independent influence on later type 2 diabetes risk rather than on cardiovascular disease. However, the results of the life course analyses presented here suggest that the increase in diabetes risk associated with high early adult BMI may be particularly important among men who have an elevated BMI in middle age; the absence of an increase in diabetes risk among men who had a high early BMI but a normal BMI in middle age suggests that the effects of high early BMI on diabetes may be reversible at least in part.
